# Impact of Natural and Synthetic Antioxidants on the Stability of High-Density Polyethylene

**DOI:** 10.3390/polym17172364

**Published:** 2025-08-30

**Authors:** Abdullah F. Alrashoudi, Hafizh Insan Akmaluddin, Maher M. Alrashed, Othman Y. Alothman

**Affiliations:** 1 Department of Chemical Engineering, King Saud University, Riyadh 12372, Saudi Arabia; 442106633@student.ksu.edu.sa (A.F.A.); 445108496@student.ksu.edu.sa (H.I.A.); mabdulaziz@ksu.edu.sa (M.M.A.); 2 Technology and Innovation, Saudi Basic Industries Corporation (SABIC), Riyadh 11422, Saudi Arabia

**Keywords:** high-density polyethylene, antioxidant, vitamin E, Irganox 1010, thermal degradation, mechanical properties, molecular weight

## Abstract

High-Density Polyethylene (HDPE) plays a crucial role in the life of every human being due to its properties such as chemical resistance, light weight, and ease of forming, among others. Its usage ranges from bottles for beverages and other liquids, to pipes, wire and cable insulation, and prosthetics. As it undergoes several thermal cycles during its life cycle, it is essential to maintain its qualities, even after undergoing thermal and thermo-oxidative degradation. Here, various dosages of synthetic (Irganox 1010) and natural (vitamin E) antioxidants are added to HDPE formulations to study their impacts on HDPE stability. The antioxidants are mixed physically with HDPE before the mixtures are melt-mixed three times to represent their life cycles. Samples are taken after each time and used to analyze the molecular weight distribution, rheological behavior, mechanical properties, and thermal stability. The results show that vitamin E is superior to Irganox 1010 in these tests, as vitamin E performance exceeds that of Irganox 1010, even at lower doses. The only drawback of using vitamin E is the yellow color it causes, which may necessitate the addition of another additive to enhance the color stability of HDPE in color-sensitive applications.

## 1. Introduction

Today, almost all our lives revolve around polymer materials. The high performance of such materials mainly causes this dependency. They are easily mass-produced, lightweight, and durable, and can be tailored to almost all the needs of humanity. High-density polyethylene (HDPE) is one of the most prominent polymer materials, accounting for around 51.33 million tons [1] or 13% [2] of global plastic production, depending on the source. HDPE has a vast field of applications, from milk and oil bottles [3] to pipes [4], wire insulation [5], and prosthetics [6]. Due to this wide range of applications, the polyethylene products available on the market are rarely pristine; instead, they are modified with additives to achieve the desired properties [7] depending on the application. Additives such as plasticizers, colorants, and antistatic agents are added to achieve key characteristics of plastics such as Izod impact, tensile strength, elongation at break, and color.

**Figure 1:**
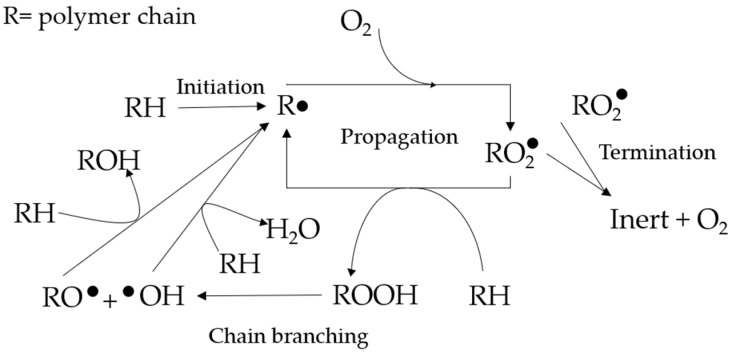
Proposed autooxidation mechanism for polymers (R = polymer chain; H = most labile hydrogen) [13].

Different end products necessitate different processing techniques. Polymer processing techniques include injection molding, blow molding, rotational molding, and extrusion, among others [8]. Polymer processing typically begins with the physical mixing of the polymer with additives. Then, the mixture is melt-mixed into pellets, so it is easier to store or transport. However, this step exposes the polymer to thermal and mechanical stresses, which cause degradation of the polymer product. This degradation is due to the polymer chains undergoing thermo-oxidative degradation at the molecular level. Under thermal and mechanical stress, free radicals are formed during the initiation stage and react with the polymer chains in the propagation stage, cutting them into weaker, lower molecular-weight chains. The propagation stage is mainly dependent on the removal of hydrogen. A polymer with a lower carbon–hydrogen bond strength will form more stable radicals, making it more susceptible to oxidation [9]. The propagation reaction will continue to occur, ranging from 800 to 17,500 cycles [10], until the radicals are deactivated in the termination stage. These reactions are simplified in Figure 1 and have been studied further in more detail [11]. Although the propagation step has been the subject of further scrutiny by scientists [12], it remains one of the simplest and most representative models of polymer degradation.

To prevent these reactions, antioxidants are added as additives to the polymer formulations. Primary antioxidants act as scavengers, reacting with free radicals in the propagation steps, while secondary antioxidants react with peroxide radicals. Both antioxidants turn the radicals from chain-cutting substances into less reactive specimens [14]. Antioxidants are essential for the polymers to maintain their properties, even after undergoing stresses. Numerous studies have been performed to study the impact of various antioxidants (for example, quercetin [15], caffeic acid, naringin, gallic acid [16], and black and green tea extract [17]) on the qualities of polymers. Currently, one of the most common [18] and effective [19] antioxidants used is Irganox 1010, which is an example of a synthetic antioxidant. However, due to environmental [20,21] and health concerns [22], there is urgency to replace them with natural antioxidants as polymer additives, such as vitamin E [23]. Previous works studied the usage of natural antioxidants for polyethylene [24,25] while also comparing the performance of natural and synthetic antioxidants in different settings, such as for maintaining food [26] and fuel quality [27].

In this work, the performances of the two antioxidants on HDPE were compared by considering the life cycle of HDPE. The polymer undergoes at least three stages of stress during its life cycle. In addition to the additive mixing process, further processing of the pellet into the desired shape exposes it to the second stress. Finally, as the polymer can be recycled into raw material after its initial use, it is exposed to the third stress. More stress can be detrimental to the qualities of the polymer. In addition, since antioxidants are known to cause discoloration in the form of yellowing [28] to the polymer product, this study added polyethylene glycol (PEG) to determine its impact in terms of preventing the discoloration of the polymer. Preliminary experiments are also performed to determine the compatibility of PEG with HDPE, Irganox 1010, and vitamin E.

## 2. Materials and Methods

### 2.1. Materials

The additives used in this study, such as antioxidants Irganox 1010 (molecular weight 1178 g/mole), vitamin E (formula C_29_H_50_O_2_), and polyethylene glycol (PEG 6000), were supplied by BASF (Ludwigshafen, Germany). The high-density polyethylene (density of 0.953 g/cm^3^) was supplied by SABIC (Riyadh, Saudi Arabia).

### 2.2. Method

#### 2.2.1. Sample Formulation and Mixing

All formulations were prepared manually using a high-precision scale (0.001 g) manufactured by Taishi (Jiaxing, China). Then, using a Henschel 5 kg laboratory mixer (Zeppelin System, Rödermark, GermanyZeppelin), all samples were dry mixed at room temperature and 800 RPM for 160 s to ensure complete homogenization for each formulation.

The formulation followed the Design of Experiment Methods [29], using a full factorial design. Three parameters were selected: the dosage of Irganox 1010 (in ppm), the dosage of vitamin E (ppm), and the number of passes. Each parameter has three levels (minimum, medium, and maximum). Thus, a total of 3 × 3 × 3 experiments were conducted. The levels and values of the parameters are presented in Table 1. The detailed formulations are shown in Table 2. Formulations 1–27 were used to study the impact of antioxidants, while formulations 28–36 were used to study the impact of PEG on the color stability of the polymer.

#### 2.2.2. Melt Mixing

Each formulation went through melt mixing by a twin-screw extruder manufactured by Thermo Fisher, Waltham, MA, USA (model PTW24/40 MC) with a screw diameter of 24 mm, 40 L/D, and 10 mixing zones. The temperature profile was 120–215 °C, with a 200 rpm screw speed. Melt mixing was repeated three times per formulation (multipass), and samples were collected after each pass. The first pass represents compounding processing by the resin supplier (such as virgin polymer extrusion). The second pass represents conversion processing (such as new article blow molding). The third pass represents the mechanical recycling of the material or reprocessing at converters. 

#### 2.2.3. Compression Molding

A Collin compression molding machine (P 300 S, Collin, Maitenbeth, Germany) was used to produce mechanical testing samples. Pellets were compressed into sheets at 180 °C for 10 min (the first 5 min at 5 bar and the next 5 min at 25 bar), then allowed to cool at a rate of 15 °C per min.

### 2.3. Characterization

#### 2.3.1. Gel Permeation Chromatography

A gel permeation chromatograph (GPC 2000, Waters Alliance, Milford, MA, USA) equipped with a differential refractive index detector was used to measure the average molecular weight (MW), number average (Mn), and polydispersity of prepared samples. To dissolve the samples, 1,2,4-trichlorobenzene stabilized by butylated hydroxytoluene was used.

#### 2.3.2. Melt Flow Index

Following the ISO 1133 standard [30], samples were placed into the melt-flow apparatus barrel and extruded through the die with specified dimensions and under a prescribed set of conditions. At 190 °C and 2.16 kg load, an extrusion plastometer (Aflow, ZwickRoell, Ulm, Germany) was used to measure melt flow characteristics.

#### 2.3.3. Dynamic Mechanical Analysis

A dynamic mechanical analysis test was conducted to measure the rheological properties of polymers. Samples were tested at a constant temperature with an increasing shear rate while recording complex viscosity as a response using a low-shear rheometer (ARES G2, TA Instruments, New Castle, DE, USA) as per ASTM D4440-15 [31].

#### 2.3.4. Tensile Properties

Following the ASTM D638-14 standard [32], compression-molded samples were tested at room temperature (23 °C). This test was used to measure the tensile strength at yield and break, as well as the elongation in both states (yield and break). A Zwick/Roell Z010 universal testing machine equipped with contact extensometer arms for strain measurements was used. A pre-load of 0.1 MPa with a gage length of 50 mm, and test speed of 1 mm/min for Young’s modulus (and 50 mm/min for other properties) were used.

#### 2.3.5. Notched Izod Impact Test

The notched Izod impact test is mainly used to measure the toughness. Following standard ASTM D256-24 [33], compression-molded samples were tested at room temperature (23 °C). Zwick/Roell HIT50P was used as an impact tester. Furthermore, test specimen dimensions were 64 × 12.7 × 3.2 mm, with 10.2 mm remaining depth following type A specifications as per ASTM D256.

#### 2.3.6. Differential Scanning Calorimetry (DSC)

This test was conducted using a Q2000 instrument (TA Instruments, New Castle, DE, USA) and followed the ASTM D3418-21 standard [34]. The sample was heated at a rate of 10 °C per minute to 200 °C, then cooled and heated again at the same rate. Results were captured in the second heating.

#### 2.3.7. Color Measurement

To measure samples’ color variation, pellets of each formulation were tested three times using a colorimeter (ColorFlex EZ, HunterLab, Reston, VA, USA) following the ASTM D6290-19 standard [35].

#### 2.3.8. Statistical Analysis

To evaluate the impact of each independent variable (concentration of Irganox 1010, concentration of vitamin E, and number of passes during the life cycle) on the characterization/test result, a three-way ANOVA was performed using Python 3.12.11 on Google Colab platform, last accessed on 29 July 2025. The impact of each independent variable, along with the two-way interaction between variables, was measured. The higher order interactions of the three independent variables were assumed to be negligible, since they correspond to the higher order of derivatives in the Taylor expansion [36]. The α value was set at 0.05. The complete results of the ANOVA are presented in Appendix A.

## 3. Results and Discussions

### 3.1. Molecular Weight and Antioxidant Mechanism

**Figure 2:**
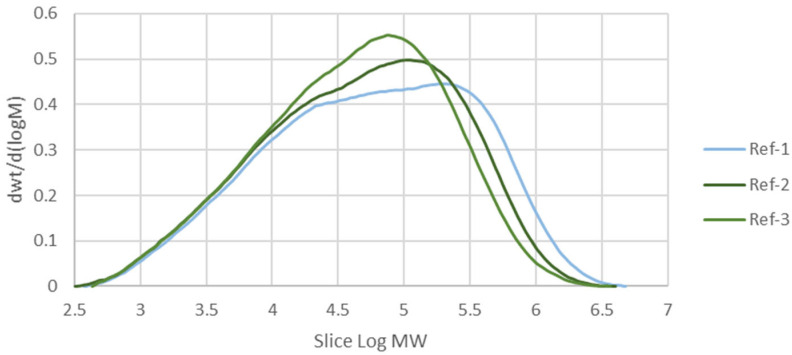
Molecular weight distribution (MWD) of the reference samples.

The average molecular weights (MW) of all the formulations are presented in Table 3. As all polymers are macromolecules, rather than a single figure, they are more accurately described by the Molecular Weight Distribution (MWD). The MWD curve being skewed to the right indicates that the polymer of concern has more fractions with high molecular weight. Figure 2 shows the MWD of the reference samples without additives, with average molecular weight values of 191,991, 141,649, and 115,091. It can be seen that the more the sample is processed (as shown by the number of passes), the less the high-molecular-weight fraction is present inside it. The processing machine applies thermal and oxidative stress to the polymer to soften it and allowing it to be shaped into the desired product. However, the same stress also broke the polymer chain and induced radical formation, which led to more broken chains, as shown in Figure 1. Hence, the MWD aligned more to the center and left as the polymer was more processed. This phenomenon is similar to what happens to LLDPE [37] and different grades of HDPE [38].

**Figure 3:**
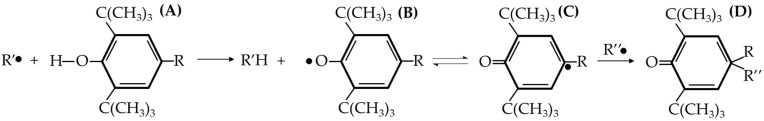
A general proposed mechanism of Irganox 1010 as an antioxidant. (**A**) The structure of Irganox; (**B**) the radical produced after it scavenges radicals; (**C**) the quinonoid radical structure from (**B**); (**D**) the inactivated radical of (**C**) after scavenging polymer radicals and stopping the propagation of polymer degradation [39]. Reprinted with permission from Ref. [39]. Copyright 2016 Wiley Periodicals.

**Figure 4:**
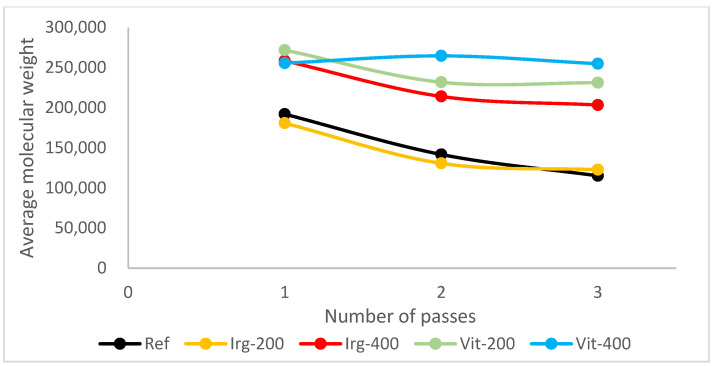
MWD of the reference sample compared with samples with antioxidants.

The first antioxidant that was used was Irganox 1010. It belongs to the hindered phenolic category and acts as a free radical scavenger. By reacting with free radicals produced by thermal and oxidative stress, it “sacrificed” itself and turned into radicals. However, Irganox 1010 has high stability due to the aromatic ring resonance and a structure that causes the hydroxyl branch to be “hindered” [37]. So, the radical derived from the antioxidant has less reactivity; thus, it will not react with other stable polymer chains. It was more likely to react with the polymer radicals and stop the propagation step in the degradation. Figure 3 shows the mechanism of Irganox 1010 as a radical scavenger. The results show that as the Irganox 1010 dosage in plastic increases, the retention of the molecular weight of the plastic samples improves. Figure 4 shows the molecular weight of specimens after each pass of the reference samples and the samples with Irganox 1010.

**Figure 5:**
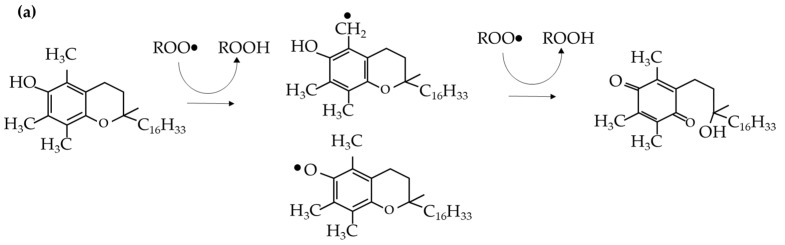

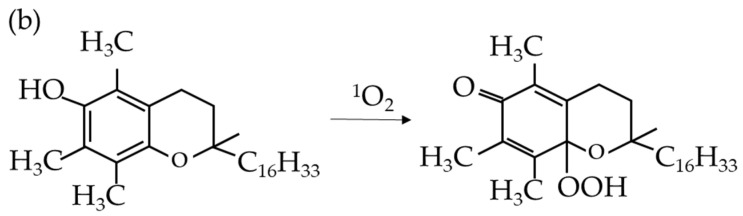
(**a**) Radical scavenging mechanism of vitamin E with quinone intermediate; (**b**) Oxygen radical stabilization mechanism of vitamin E [7]. Reprinted with permission from Ref. [7]. Copyright 2017 De Gruyter.

The second antioxidant studied in this experiment is vitamin E. Vitamin E, in its most active form (α-tocopherol), is a well-known antioxidant that is often taken as a supplement to the human diet, as it confers many benefits [40] and its deficiency causes some health-related problems [41]. It also acts as an antioxidant to polymers, albeit with a different mechanism compared to Irganox 1010. The hydroxyl branch in vitamin E exists in the chromane ring in Vitamin E [42]. The hydrogen of this hydroxyl group is donated to radical molecules to stabilize reactive organic species and help reduce the chain scission process. Figure 5a shows the action mechanism of vitamin E [7].

**Figure 6:**
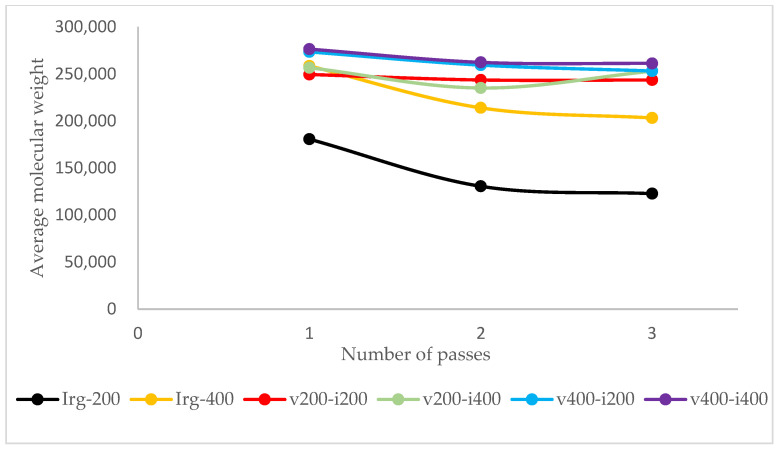
Average molecular weight of each pass for several formulations of the sample.

The experimental results show that introducing vitamin E to the samples resulted in better retention of its molecular weight, regardless of the number of passes or dosage, as illustrated in Figure 4. The better performance of vitamin E over Irganox 1010 was confirmed by ANOVA, as shown in Table A1. Moreover, as vitamin E is not as sterically hindered as the Irganox 1010 molecule, it allows for more mechanisms to react with free radicals, such as by responding directly to oxygen radicals (Figure 5b). The performance of vitamin E is compared with that of Irganox 1010 at the same dosage in Figure 4. As vitamin E and Irganox 1010 have good compatibility, vitamin E can also be used as an additive in plastic formulations with Irganox 1010, as shown in Figure 6.

### 3.2. Melt Flow Index

Table 3 shows that MFI increased as the reference polymer sample went on each pass: 6.8 g/10 min after the first pass and jumped to 31.3 g/10 min after three passes. The increase in MFI is caused by the lower molecular weight due to the degradation process that occurs during heating [43]. The lower molecular weight will reduce the viscosity, making the melt easier to flow, and thus increasing the melt flow index.

With the addition of 200 ppm of Irganox 1010, a slight improvement was observed, but the MFI increase remained significant. ANOVA also showed that Irganox 1010 does not significantly impact the melt flow index. However, 200 ppm of vitamin E was sufficient to retain the MFI of the sample, even after the third pass. The performance of vitamin E in controlling MFI is in agreement with a previous study [43].

**Figure 7:**
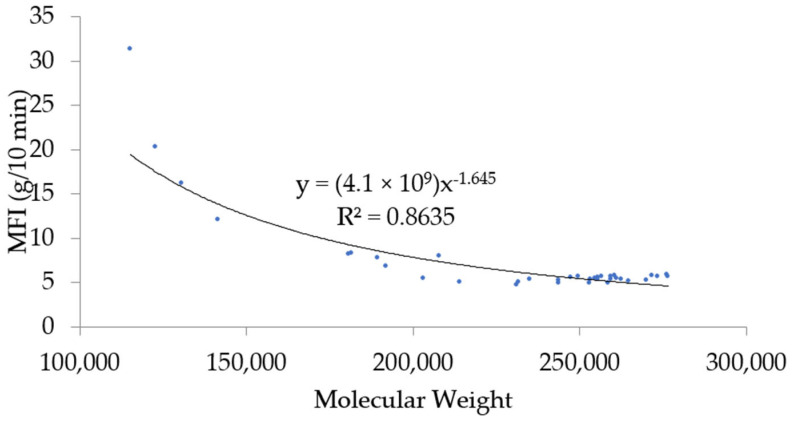
Melt flow index as a function of molecular weight according to Bremner et al. [44].

In addition, the relationship between the molecular weight and melt flow index can be correlated with an empirical equation formulated by Bremner et al. [44]. The relationship is shown in Figure 7.

### 3.3. Dynamic Mechanical Analysis

**Figure 8:**
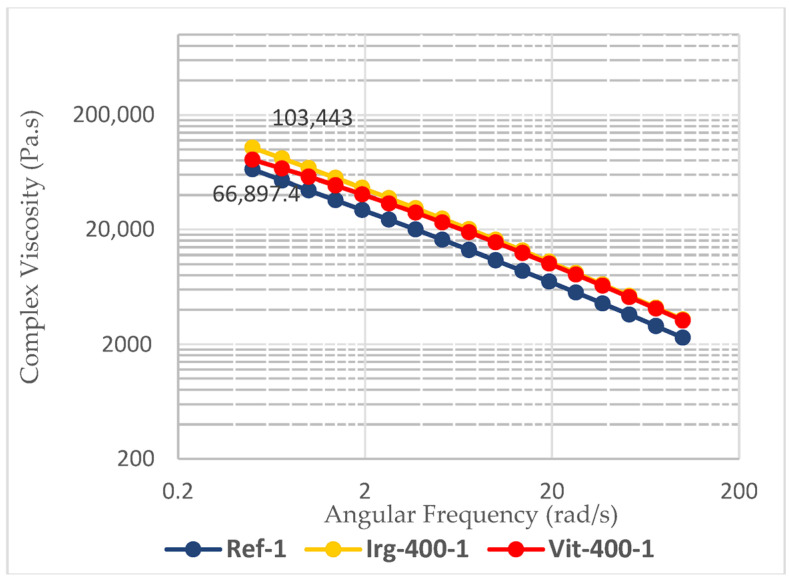
Shear thinning behavior of the 1st pass of reference samples and samples with 400 ppm antioxidants.

**Figure 9:**
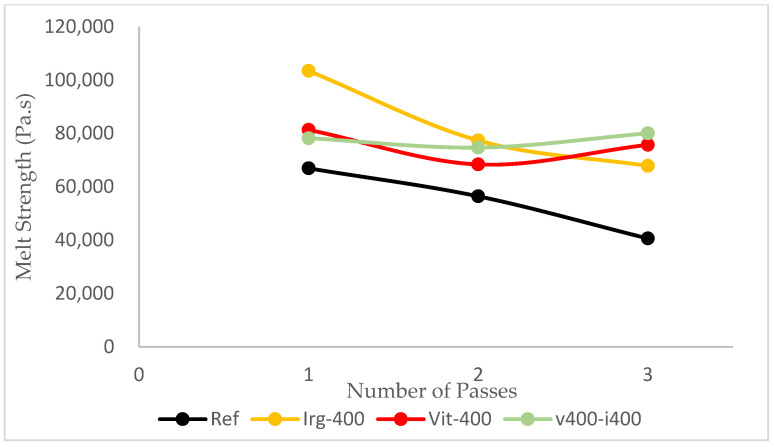
Melt strength of the reference sample and samples with 400 ppm of Irganox 1010, 400 ppm of vitamin E, and 400 ppm of both antioxidants.

In the dynamic mechanical analysis, the shear thinning behavior was exhibited by all three passes of the reference and all formulations, as shown in Table 3 (and some are shown in Figure 8), which is an essential property for the ease of processing. The shearstress applied by the equipment will break the intermolecular forces of the polymer chains and allow them to flow more easily. However, the value of the melt strength should also be considered. The melt strength is determined based on the complex viscosity at a low shear rate (angular frequency of 0.5 rad/s). Without any antioxidants, the complex viscosity of the reference samples drops by about 40% from 66,897.4 Pa.s in the first pass to 40,640.1 Pa.s in the third pass. This leads to sagging of the parison during the blow-molding process. Such sagging leads to less control over the wall thickness of the blow-molded bottle [45]. With respect to this parameter, the addition of Irganox 1010 slightly reduced the drop in melt strength from 39.25% to 34.44% after the third pass. By using 400 ppm of vitamin E (7% drop) or combining 400 ppm of Irganox 1010 and 400 ppm of vitamin E (2% drop), the melt strength of HDPE was relatively more maintained, as shown in Figure 9.

### 3.4. Izod Impact Strength

The Izod impact strength test measures the impact resistance of the samples. As discussed in a previous study [46], impact resistance is related to the molecular weight. A polymer with a higher molecular weight can withstand a higher impact due to the presence of longer polymer chains that intertwine with each other, providing free volume inside and acting like a “foam”. Therefore, the incoming force is absorbed and dispersed in different directions, preventing it from breaking the polymer bond and forming a notch physically [47]. Table 4 shows the impact strength of the reference samples, which showed evident deterioration after each pass. It dropped from 515.2 J/m to 152.4 J/m, which represents a 70% drop in impact strength.

**Figure 10:**
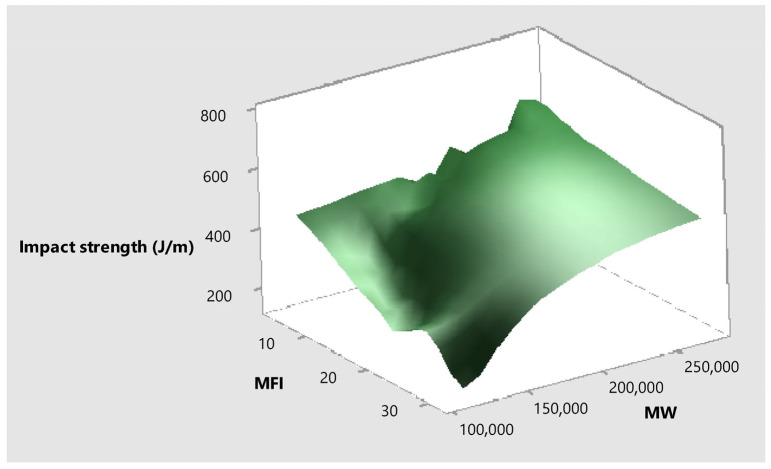
Surface plot of the relationship between impact strength, MFI, and MW.

The addition of Irganox 1010 at doses of 200 and 400 ppm only improved the impact strength of the HDPE by a small margin, as it still dropped by 49% and 41% from the initial impact strength after the third pass; thus, they were categorized as insignificant impacts in the ANOVA. On the other hand, vitamin E alone provided better results at both dosages (9% drop in 200 ppm and 1% drop in 400 ppm). Combining vitamin E with a formulation with 400 ppm of Irganox 1010 also improved the impact strength (15% drop for 200 ppm vitamin E and almost 0% drop with the addition of 400 ppm vitamin E). Figure 10 shows the relationship between the impact strength, molecular weight, and melt flow index. It is evident that as the MFI decreases due to a higher molecular weight, the impact strength increases. A similar relation between MFI, molecular weight, and impact strength was observed in a previous study [46].

### 3.5. Tensile Properties

Another critical property for blow-molding HDPE grades is tensile strength, which is linked to the top-load test of blow-molded articles. The top-load test is one of the critical quality properties of bottle-shaped producers. Table 4 shows that the tensile strength at yield is retained in all formulations and is only affected by the number of passes. The value of Young’s Modulus of reference samples increases by 11.22% between the 1st and 3rd pass (from 1390 to 1546 MPa). The value of elongation at break drops from 410% at the first pass to 27.47% after the third pass. The longer polymer chains in the first pass allow the sample to be stretched farther as the polymer chains straighten. The intermolecular forces between chains are also weak compared to the chain bond, so chain slippage can happen [46], allowing the sample to elongate. Samples with the addition of Irganox 1010 still experience more than 59% drops in elongation at break after the third pass at both dosages. Meanwhile, vitamin E helped retain the elongation at break at both dosages, limiting the drop in the elongation at break after the third pass to 3.16% and 6.05% for 200 ppm and 400 ppm, respectively. These results are in agreement with the Izod impact data discussed in the previous section, where vitamin E samples exhibited superior performance (higher impact strength and higher elongation) over the Irganox 1010-stabilized samples.

### 3.6. Differential Scanning Calorimetry

The measurement of the crystallinity and thermal characteristics was performed using DSC on the samples to be used for blow molding after the first pass. The samples’ thermal history was erased by heating the specimens twice to eliminate inconsistencies within the crystalline structure caused by variations in solidification after pelletizing. Two tests were performed per sample, and the results are shown in Table 3. ANOVA result shows that although the melting points across the samples are similar, there is a significant impact from the number of passes. On the other hand, the crystallinities of the samples did not change significantly as a result of the addition of either antioxidant or changing the number of passes. This may be due to the higher chain scissions after each pass. The loss of higher molecular-weight chains and the creation of shorter ones causes a change in the crystallinity of the polymer [48].

### 3.7. Color Measurement

In Table 3, it is shown that each pass through the melt mixer slightly increased the yellowness of the reference samples, albeit categorized as insignificant according to ANOVA. Yellowness is caused by two reasons [24]. The first reason is the polymer. The yellowness is caused by the chiral, optically active supramolecules that were formed on the surface of the polymer due to heat and UV degradation [49]. The second reason is the activity of the antioxidant as an additive itself. The phenolic antioxidant scavenges the radicals into hydroxyl groups, producing quinoidal compounds as side products [24]. Samples enhanced with vitamin E exhibited even more yellowness due to the less steric hindrance on the phenol in vitamin E [50], which made it more prone to react with residues of metal ion catalyst from the polymerization process, thereby causing more yellowness compared to Irganox 1010. Higher yellowness decreases the suitability of HDPE in specific applications that require white or transparent color, such as food, beverages, and pharmaceutical packaging [51,52].

To solve this problem, polyethylene glycol (PEG) was added to the formulation. The addition of PEG resulted in a decrease in the yellowness index for the reference and samples with antioxidants. The hydroxyl group of glycols competes with antioxidants to react with catalyst remains, preventing the production of discoloration substances [53]. PEG can also react with the quinoidal compound present in the sample due to the radical scavenging activities of the antioxidants, similar to how PEG is degraded by quinoidal substances in its biodegradation by fungi [54,55,56]. Based on the mechanical properties test of sample numbers 28–36, which showed relatively similar results, we hypothesized that PEG is compatible with HDPE and the currently used antioxidants (Irganox 1010 and vitamin E). Further compatibility tests, along with the exact nature and mechanism of PEG interaction with HDPE, Irganox 1010, and vitamin E, could be explored in further studies. 

## 4. Conclusions

This study aimed to compare the performance of natural antioxidants (vitamin E) with that of synthetic antioxidants (Irganox 1010) in protecting high-density polyethylene from degradation in response to various stresses during its lifetime. As a reference, HDPE was passed through a melt-mixer three times to mimic the life cycle of plastics. The first pass symbolizes pellet processing, the second pass is for parison/final processing, and the third phase is the mechanical recycling stage. The HDPE underwent degradation due to the chain scission of the polymer, resulting in a decrease in molecular weight and a skewing of its distribution to the left.

The result of this study shows that the addition of an antioxidant as an additive will reduce the impact of thermal stress on the polymer’s properties. Further experiments demonstrated that vitamin E, as a natural antioxidant, outperforms Irganox 1010 in specific parameters due to the less hindered structure of its molecules. Results of ANOVA also confirm that vitamin E is a more impactful factor than Irganox 1010 in terms of the properties of HDPE and the number of passes. Vitamin E is more effective in helping HDPE to maintain its molecular weight, melt flow index, and mechanical properties such as tensile strength and elongation at break. Vitamin E can also be used as an additive for synthetic antioxidants, as adding vitamin E to formulations with Irganox 1010 maintained or improved the properties of the polymer. However, vitamin E caused worse discoloration of the final product compared to Irganox 1010. So, the usage of vitamin E as an antioxidant should be limited to HDPE products that do not include color as a quality requirement, such as biomaterials [57] and packaging for radiative sterilization of medical equipment [58]. To reduce the discoloration of the final product, 400 ppm of polyethylene glycol was added to the formulations, either with Irganox 1010, vitamin E, or both, to reduce the yellowing of the HDPE. Since the preliminary result shows that PEG reduces yellowing and does not negatively impact the desired properties of HDPE, further studies are necessary to confirm these findings.

## Figures and Tables

**Table 1:** Levels of each parameter tested during this research.

Parameter	No. of Levels	Values
Concentration of Vitamin E	3	0, 200, and 400 (ppm)
Concentration of Irganox 1010	3	0, 200, and 400 (ppm)
Number of Passes	3	1 to 3

**Table 2:** Details on each formulation used in this research.

Formulation No.	Formulation Name	Number of Passes	Polymer	Concentration of Irganox 1010 (ppm)	Concentration of Vitamin E (ppm)	Concentration of PEG (ppm)
1	Ref-1	1	PE	0	0	0
2	Ref-2	2	PE	0	0	0
3	Ref-3	3	PE	0	0	0
4	Irg-200-1	1	PE	200	0	0
5	Irg-200-2	2	PE	200	0	0
6	Irg-200-3	3	PE	200	0	0
7	Irg-400-1	1	PE	400	0	0
8	Irg-400-2	2	PE	400	0	0
9	Irg-400-3	3	PE	400	0	0
10	Vit-200-1	1	PE	0	200	0
11	Vit-200-2	2	PE	0	200	0
12	Vit-200-3	3	PE	0	200	0
13	v200-i200-1	1	PE	200	200	0
14	v200-i200-2	2	PE	200	200	0
15	v200-i200-3	3	PE	200	200	0
16	v200-i400-1	1	PE	400	200	0
17	v200-i400-2	2	PE	400	200	0
18	v200-i400-3	3	PE	400	200	0
19	Vit-400-1	1	PE	0	400	0
20	Vit-400-2	2	PE	0	400	0
21	Vit-400-3	3	PE	0	400	0
22	v400-i200-1	1	PE	200	400	0
23	v400-i200-2	2	PE	200	400	0
24	v400-i200-3	3	PE	200	400	0
25	v400-i400-1	1	PE	400	400	0
26	v400-i400-2	2	PE	400	400	0
27	v400-i400-3	3	PE	400	400	0
28	Irg-400-PEG-1	1	PE	400	0	150
29	Irg-400-PEG-2	2	PE	400	0	150
30	Irg-400-PEG-3	3	PE	400	0	150
31	v400-i400-PEG-1	1	PE	400	400	150
32	v400-i400-PEG-2	2	PE	400	400	150
33	v400-i400-PEG-3	3	PE	400	400	150
34	v400-PEG-1	1	PE	0	400	150
35	v400-PEG-2	2	PE	0	400	150
36	v400-PEG-3	3	PE	0	400	150

**Table 3:** Physical properties of HDPE with various additive formulations.

Formulation Name	MW	% of Change from 1st Pass	MFI (g/10 min)	% of Change from 1st Pass	Melt Strength (Pa.s)	% of Change from 1st Pass	Crystallinity (%)	Melting Point (°C)	Color (b-Value)
Ref-1	191,991	-	6.79	-	66,897.4	-	68.75	130.4	−1.1
Ref-2	141,649	−26.22	12.04	77.32	56,409.3	−15.68	58.45	130.6	−0.3
Ref-3	115,091	−40.05	31.3	360.97	40,640.1	−39.25	51.3	136.1	0.16
Irg-200-1	180,731	-	8.21	-	70,690.1	-	61.2	130.7	−0.95
Irg-200-2	130,614	−27.73	16.22	97.56	46,159.6	−34.70	57.25	130.3	−0.5
Irg-200-3	122,713	−32.10	20.3	147.26	44,000.0	−37.76	59.1	129.9	0.3
Irg-400-1	258,469	-	4.92	-	103,443	-	60.4	130.2	−1.01
Irg-400-2	213,998	−17.21	5.02	2.03	77,374.6	−25.20	40.25	133.4	−0.4
Irg-400-3	203,260	−21.36	5.4	9.76	67,816.8	−34.44	44.15	138.3	0.76
Vit-200-1	271,788	-	5.77	-	55,153.4	-	58	129.9	7.6
Vit-200-2	231,764	−14.73	5.01	−13.17	65,798.2	19.30	58.55	129.9	4.8
Vit-200-3	231,207	−14.93	4.7	−18.54	58,516.1	6.10	37.4	136.4	4.4
v200-i200-1	249,505	-	5.6	-	76,354.4	-	56.25	131.2	4.02
v200-i200-2	243,617	−2.36	5.2	−7.14	72,591.1	−4.93	56.55	131.1	4.5
v200-i200-3	243,665	−2.34	4.9	−12.50	68,519.5	−10.26	57.9	130.8	6.7
v200-i400-1	256,631	-	5.65	-	78,902.2	-	56.25	130.1	3.4
v200-i400-2	235,056	−8.41	5.3	−6.19	77,735.1	−1.48	58	130.5	4.6
v200-i400-3	253,072	−1.39	4.9	−13.27	50,340.9	−36.20	59.25	130.6	5.6
Vit-400-1	255,547	-	5.52	-	81,341	-	59.945	129.8	8.6
Vit-400-2	264,646	3.56	5.1	−7.61	68,385.9	−15.93	56.3	131.0	8.4
Vit-400-3	254,776	0.30	5.4	−2.17	75,705.1	−6.93	59.75	130.8	9.5
v400-i200-1	273,529	-	5.65	-	82,914.6	-	58.7	130.7	8.9
v400-i200-2	259,465	−5.14	5.35	−5.31	72,063	−13.09	57.75	130.5	8.2
v400-i200-3	253,196	−7.43	5.3	−6.19	78,491.7	−5.33	58.45	130.5	9.2
v400-i400-1	276,384	-	5.63	-	78,188	-	58.35	129.8	12.1
v400-i400-2	262,368	−5.07	5.37	−4.62	74,700.4	−4.46	58.4	130.9	8.36
v400-i400-3	261,212	−5.49	5.4	−4.09	80,037.9	2.37	58.9	130.8	10.1
Irg-400-PEG-1	181,589	-	8.33	-	65,291.2	-	59.75	130.5	−0.7
Irg-400-PEG-2	189,411	4.31	7.8	−6.36	63,268	−3.10	52.95	130.3	−0.45
Irg-400-PEG-3	207,835	14.45	8.01	−3.84	64,865	-0.65	58.5	130.4	−0.4
v400-i400-PEG-1	247,305	-	5.57	-	75,778.6	-	60.7	130.6	12.5
v400-i400-PEG-2	255,467	3.30	5.33	−4.31	76,537.2	1.00	59	130.3	6.2
v400-i400-PEG-3	260,584	5.37	5.7	2.33	76,432.1	0.86	59.85	130.4	7.5
v400-PEG-1	276,271	-	5.84	-	72,303.3	-	58.5	129.9	6.6
v400-PEG-2	270,180	−2.20	5.24	−10.27	73,905.6	2.22	61.05	130.3	9.1
v400-PEG-3	259,343	−6.13	5.6	−4.11	68,169.6	−5.72	60.1	130.5	7.96

**Table 4:** Mechanical properties of HDPE with various additive formulations.

Formulation Name	Izod Impact (J/m)	% of Change from 1st Pass	Tensile Strength at Yield (MPa)	% of Change from 1st Pass	Young Modulus (MPa)	% of Change from 1st Pass	Tensile Elongation at Break (%)	% of Change from 1st Pass
Ref-1	515.2	-	25.6	-	1390	-	410	-
Ref-2	290.5	−43.61	27	5.47	1500.8	7.97	49.8	−87.85
Ref-3	152.4	−70.42	27.6	7.81	1546	11.22	27.4	−93.32
Irg-200-1	392.2	-	25.9	-	1400	-	292	-
Irg-200-2	238.7	−39.14	27.8	7.34	1548.8	10.63	35	−88.01
Irg-200-3	201	−48.75	26.3	1.54	1532	9.43	31	−89.38
Irg-400-1	584.5	-	25.6	-	1360	-	552.5	-
Irg-400-2	539.8	−7.65	26.5	3.52	1424.8	4.76	420	−23.98
Irg-400-3	347.3	−40.58	27.4	7.03	1503	10.51	222.5	−59.73
Vit-200-1	519.1	-	25.8	-	1372	-	526.7	-
Vit-200-2	553.5	6.63	27.1	5.04	1505	9.69	458	−13.04
Vit-200-3	471.8	−9.11	26.8	3.88	1495	8.97	510	−3.16
v200-i200-1	537.2	-	25.5	-	1352	-	542	-
v200-i200-2	595.7	10.89	25.3	−0.78	1556	15.09	516	−4.80
v200-i200-3	464.7	−13.50	26.8	5.10	1491	10.28	523.3	−3.44
v200-i400-1	551	-	25.9	-	1391	-	436.7	-
v200-i400-2	588.4	6.79	28	8.11	1562	12.29	428	−1.98
v200-i400-3	468.8	−14.92	26.7	3.09	1498	7.69	552	26.41
Vit-400-1	617.4	-	25.6	-	1363	-	562	-
Vit-400-2	663.6	7.48	27.5	7.42	1459.8	7.10	448	−20.28
Vit-400-3	614.1	−0.53	26.7	4.30	1490	9.32	528	−6.05
v400-i200-1	776.3	-	27	-	1503	-	470	-
v400-i200-2	560.3	−27.82	27.3	1.11	1459	−2.93	362	−22.98
v400-i200-3	535.9	−30.97	26.7	−1.11	1498	−0.33	552	17.45
v400-i400-1	550.6	-	25.4	-	1331.6	-	554	-
v400-i400-2	644	16.96	27.3	7.48	1513.6	13.67	502	−9.39
v400-i400-3	551	0.07	26.1	2.76	1446	8.59	496.7	−10.35
Irg-400-PEG-1	371	-	25.7	-	1362.2	-	216	-
Irg-400-PEG-2	415.5	11.99	26.9	4.67	1405	3.14	264	22.22
Irg-400-PEG-3	390.2	5.18	25.2	−1.95	1405	3.14	272	25.93
v400-i400-PEG-1	547.4	-	25	-	1317.6	-	443.3	-
v400-i400-PEG-2	589.6	7.71	27.4	9.60	1546.2	17.35	454	2.41
v400-i400-PEG-3	614.1	12.18	27.1	8.40	1514	14.91	527.5	18.98
v400-PEG-1	485.1	-	25.4	-	1344	-	578	-
v400-PEG-2	533	9.87	27.3	7.48	1479.6	10.09	424	−26.64
v400-PEG-3	606.3	24.98	26.8	5.51	1480	10.12	552	−4.50

## Data Availability

The original contributions presented in the study are included in the article, further inquiries can be directed to the corresponding author.

## Abbreviations

The following abbreviations are used in this manuscript:

DSC	Differential scanning calorimetry
GPC	Gel permeation chromatography
HDPE	High-density polyethylene
MFI	Melt flow index
MW	Molecular weight
MWD	Molecular weight distribution
PEG	Polyethylene glycol

## Appendix A

### Appendix A.1. ANOVA Result

Table A1 contains the results of the three-way ANOVA for vitamin E, Irganox 1010, and the number of passes.

**Table A1:** ANOVA parameters and results.

Dependent Variables	Parameter	C(Vitamin E Concentration) (A)	C(Irganox 1010 Concentration) (B)	C(Extruder_Passes) (C)	C(AxB)	C(AxC)	C(BxC)	Residual
Molecular Weight	sum_sq	40,660,615,484.963	5,112,176,817.852	4,887,268,419.185	7,249,427,183.926	2,727,185,360.593	231,342,411.037	692,318,198.519
	df	2	2	2	4	4	4	8
	F	234.924	29.537	28.237	20.942	7.878	0.668	
	PR(>F)	0	0	0	0	0.007	0.632	
Melt Flow Index	sum_sq	287.892	75.254	66.415	158.492	169.945	58.106	115.744
	df	2	2	2	4	4	4	8
	F	9.949	2.601	2.295	2.739	2.937	1.004	
	PR(>F)	0.007	0.135	0.163	0.105	0.091	0.459	
Melt Strength	sum_sq	9,823,371,593.959	2,040,345,883.352	901,136,840.613	11,162,183,127.526	788,960,163.636	152,637,737.101	323,104,911.162
	df	2	2	2	4	4	4	8
	F	121.612	25.259	11.156	69.093	4.884	0.945	
	PR(>F)	0	0	0.005	0	0.027	0.486	
Crystallinity	sum_sq	54.585	47.37	156.38	272.813	189.677	157.667	192.251
	df	2	2	2	4	4	4	8
	F	1.136	0.986	3.254	2.838	1.973	1.64	
	PR(>F)	0.368	0.414	0.092	0.098	0.192	0.255	
Melting Point	sum_sq	12.921	6.072	27.523	18.457	13.486	23.215	20.296
	df	2	2	2	4	4	4	8
	F	2.546	1.197	5.424	1.819	1.329	2.288	
	PR(>F)	0.139	0.351	0.032	0.219	0.338	0.148	
Color (b-value)	sum_sq	416.928	0.549	4.59	5.078	3.907	2.525	13.02
	df	2	2	2	4	4	4	8
	F	128.089	0.169	1.41	0.78	0.6	0.388	
	PR(>F)	0	0.848	0.299	0.569	0.673	0.812	
Izod Impact Strength	sum_sq	291,400.056	17,262.954	89,542.574	64,381.761	43,966.268	13,301.037	34,340.561
	df	2	2	2	4	4	4	8
	F	33.942	2.011	10.43	3.75	2.561	0.775	
	PR(>F)	0	0.196	0.006	0.053	0.12	0.571	
Tensile Strength at Yield	sum_sq	0.227	0.072	8.036	2.404	0.959	1.075	4.01
	df	2	2	2	4	4	4	8
	F	0.227	0.072	8.017	1.199	0.478	0.536	
	PR(>F)	0.802	0.931	0.012	0.382	0.751	0.714	
Young’s Modulus	sum_sq	5,659,780.436	1,328,043.662	63,984.916	2,661,506.569	18,542.222	3725.102	16,247.840
	df	2	2	2	4	4	4	8
	F	3431.335	805.148	38.792	806.791	5.621	1.129	
	PR(>F)	0	0	0	0	0.019	0.408	
Elongation at Break	sum_sq	442,291.500	43,017.816	79,110.527	102,683.404	115,998.507	14,373.064	22,137.829
	df	2	2	2	4	4	4	8
	F	79.916	7.773	14.294	9.277	10.48	1.299	
	PR(>F)	0	0.013	0.002	0.004	0.003	0.348	
